# Routine Antenatal Anti-D Prophylaxis in Women Who Are Rh(D) Negative: Meta-Analyses Adjusted for Differences in Study Design and Quality

**DOI:** 10.1371/journal.pone.0030711

**Published:** 2012-02-03

**Authors:** Rebecca M. Turner, Myfanwy Lloyd-Jones, Dilly O. C. Anumba, Gordon C. S. Smith, David J. Spiegelhalter, Hazel Squires, John W. Stevens, Michael J. Sweeting, Stanislaw J. Urbaniak, Robert Webster, Simon G. Thompson

**Affiliations:** 1 Medical Research Council Biostatistics Unit, Institute of Public Health, Cambridge, United Kingdom; 2 Health Economics and Decision Science, University of Sheffield, Sheffield, United Kingdom; 3 Academic Unit of Reproductive and Developmental Medicine, The University of Sheffield Medical School, Sheffield, United Kingdom; 4 Department of Obstetrics and Gynaecology, University of Cambridge, Cambridge, United Kingdom; 5 Statistical Laboratory, University of Cambridge, Cambridge, United Kingdom; 6 Centre for Bayesian Statistics in Health Economics, University of Sheffield, Sheffield, United Kingdom; 7 Academic Transfusion Medicine Unit, University of Aberdeen, Foresterhill, United Kingdom; 8 National Blood Service Sheffield Centre, National Health Service Blood and Transplant, Sheffield, United Kingdom; 9 Department of Public Health and Primary Care, University of Cambridge, Cambridge, United Kingdom; University of Sao Paulo – USP, Brazil

## Abstract

**Background:**

To estimate the effectiveness of routine antenatal anti-D prophylaxis for preventing sensitisation in pregnant Rhesus negative women, and to explore whether this depends on the treatment regimen adopted.

**Methods:**

Ten studies identified in a previous systematic literature search were included. Potential sources of bias were systematically identified using bias checklists, and their impact and uncertainty were quantified using expert opinion. Study results were adjusted for biases and combined, first in a random-effects meta-analysis and then in a random-effects meta-regression analysis.

**Results:**

In a conventional meta-analysis, the pooled odds ratio for sensitisation was estimated as 0.25 (95% CI 0.18, 0.36), comparing routine antenatal anti-D prophylaxis to control, with some heterogeneity (*I* ^2^ = 19%). However, this naïve analysis ignores substantial differences in study quality and design. After adjusting for these, the pooled odds ratio for sensitisation was estimated as 0.31 (95% CI 0.17, 0.56), with no evidence of heterogeneity (*I* ^2^ = 0%). A meta-regression analysis was performed, which used the data available from the ten anti-D prophylaxis studies to inform us about the relative effectiveness of three licensed treatments. This gave an 83% probability that a dose of 1250 IU at 28 and 34 weeks is most effective and a 76% probability that a single dose of 1500 IU at 28–30 weeks is least effective.

**Conclusion:**

There is strong evidence for the effectiveness of routine antenatal anti-D prophylaxis for prevention of sensitisation, in support of the policy of offering routine prophylaxis to all non-sensitised pregnant Rhesus negative women. All three licensed dose regimens are expected to be effective.

## Introduction

Women who are Rhesus negative require particular antenatal care and monitoring during pregnancy. If the fetus carried is Rhesus positive, there is a risk that mixing of fetal and maternal blood cells will lead to the woman becoming sensitised, which may cause fetuses in any subsequent pregnancies to suffer from haemolytic disease of the newborn [1]. Women in developed countries have long been offered targeted anti-D immunoglobulin with the aim of preventing sensitisation, after the birth of a Rhesus positive baby and after other potentially sensitising events such as miscarriage, termination of pregnancy or amniocentesis [2]. Under this policy, the incidence of haemolytic disease of the newborn was substantially reduced but was still believed to cause death in 6 out of 100 000 live births [3]. The UK National Institute for Health and Clinical Excellence (NICE) evaluated, first in 2003 and subsequently updated in 2009, the potential benefits from offering routine antenatal anti-D prophylaxis (RAADP) to all non-sensitised Rhesus negative pregnant women [4], [5]. In both evaluations, it was concluded that RAADP was cost-effective and should be delivered to all non-sensitised Rhesus negative pregnant women. The second NICE report recommended further research to compare the effectiveness of the different licensed RAADP regimens, since the available studies provided insufficient evidence to inform a treatment comparison [5]. Two of the three licensed regimens have not yet been evaluated in practice. However, published studies provide evidence on the effectiveness of dose regimens similar to the two unevaluated regimens, and on the third licensed regimen directly.

The NICE appraisals identified 10 studies which evaluated the clinical effectiveness of RAADP compared to control [6]–[15]. However, the studies were generally of poor quality and varied substantially in study design. For example, many of the studies used historical rather than concurrent controls, only one study was randomised, and there were often post-hoc exclusions and large numbers of women lost to follow-up. Across the 10 studies, there were differences in the doses and timing of administering anti-D immunoglobulin, in the obstetric characteristics of the women recruited to each study, and in the follow-up times. Differences in study design and methodological limitations make it inappropriate to use a conventional meta-analysis to combine the study results and draw overall conclusions. In the NICE appraisals, conclusions on clinical effectiveness and cost-effectiveness were therefore based primarily on a meta-analysis of only the two studies considered to be most relevant to the UK setting and of acceptable quality [12], [13].

There are two major weaknesses of conventional meta-analysis. First, by excluding trials which fall below an arbitrary threshold of quality, some of the available evidence is ignored. Second, by simply pooling the results of studies above the threshold, known biases in these studies are not accounted for. Methodological quality is assessed by the majority of systematic review authors [16], but only about 50% make use of the quality assessment, usually in sensitivity analyses or subgroup analyses [17]. In general, the primary results presented from a meta-analysis make no allowance for differences in quality or design among the studies included. It is misleading to report confidence intervals which represent only the uncertainty due to random error when systematic biases are suspected [18].

Recently, four of the authors of this paper proposed methods for performing bias-adjusted meta-analysis, enabling adjustment for differences in quality and design [19]. The advantages of bias-adjusted meta-analysis are that all available evidence can be synthesised and that the analysis allows for biases present in the studies. To exemplify the methods, the previous paper demonstrated their application to 8 of the anti-D studies [19]. However, the bias adjustment process was carried out for illustrative purposes in our earlier paper, without the involvement of experts on anti-D prophylaxis, and the results were not intended to inform clinical practice. In the present paper, we have obtained new pooled bias-adjusted results for the effectiveness of RAADP, based on a synthesis of all 10 available studies. The impact of differences in dose regimen, follow-up times and study populations was evaluated by assessors with knowledge of anti-D prophylaxis, while the impact of methodological flaws in the studies was evaluated by assessors with quantitative expertise. To inform the analysis comparing different RAADP dose regimens, we also elicited opinion on the relative effectiveness of all treatment regimens of relevance, comprising the five treatments evaluated in one or more studies and the two treatments which are licensed but as yet unevaluated. A meta-regression was performed to estimate the association between the observed effectiveness of different anti-D dose regimens and pooled opinion on the effectiveness of each regimen relative to an optimally effective treatment. This enabled estimation of the differences in effectiveness between all three licensed dose regimens of anti-D immunoglobulin, which has not been possible previously.

## Methods

### Source studies

The analyses presented included the studies identified through systematic literature searches in the two UK NICE technology appraisals of RAADP [4], [5]. The first NICE appraisal identified 10 studies which compared RAADP to control [6]–[15]. The second appraisal identified only one new relevant study, which compared intravenous against intramuscular delivery of the same dose of RAADP. Two of the original 10 studies comparing RAADP to control were excluded from the second NICE appraisal, since these evaluated doses which were unlicensed in the UK. In the primary analyses presented in this paper, all 10 studies comparing RAADP to control were included. Amongst these ten, three studies [6]–[8] by Bowman and colleagues used the same group of women as controls. The later two studies were not strictly comparative studies, since the focus in each of them was on describing a new treated group of women. However, these two studies are useful in providing evidence on the effectiveness of a single high dose of RAADP. In most analyses we included all three Bowman studies, but in a sensitivity analysis we excluded the later two.

### Bias-adjustment method

A recently proposed meta-analytic method [19] allows adjustment for both methodological limitations (internal biases) in the set of studies to be combined and differences in study design relative to the research question of interest (external biases). The method was implemented using the following steps, which we discuss further below: define the target setting; describe an idealised version of each available study; identify internal and external biases; elicit expert opinion on the magnitude and uncertainty of the biases; perform a bias-adjusted meta-analysis. To demonstrate the methods, we use the study by Trolle [15], which evaluated a dose of 1500 IU anti-D immunoglobulin at 28 weeks' gestation, in non-sensitised pregnant Rhesus negative women attending a Danish hospital.

We use the term “target setting” to refer to the research question of interest. In our evaluation of the effectiveness of RAADP, the target setting reflects the objectives of the first NICE appraisal in terms of the population to which the findings will be applied, the intervention and control policies being compared, and the outcome of interest. These were defined as:

- Population: Non-sensitised pregnant Rhesus negative women in the UK
- Intervention: Dose of 500 IU anti-D immunoglobulin offered intramuscularly at 28 and 34 weeks' gestation, in addition to control antenatal care
- Control: Anti-D immunoglobulin offered postpartum and after potentially sensitising events during pregnancy, according to 2002 UK policy
- Outcome: Prevention of Rhesus sensitisation which would affect a subsequent pregnancy

The next step in the bias-adjustment process was to define an idealised version of each study included in the meta-analysis. The idealised study is an imagined repeat of the original study, in which the design would be modified to eliminate all sources of internal bias (i.e. methodological limitations). The protocol for an idealised study is a tool which helps us identify internal and external biases in the original study, and the design need not be practically possible. As an example, we present the protocol for an idealised version of Trolle [15].

- Population: Non-sensitised Rhesus negative women delivered of Rhesus positive babies at Kolding Hospital, Denmark
- Intervention: Dose of 1500 IU anti-D immunoglobulin given at 28 weeks' gestation, in addition to control antenatal care
- Control: Dose of at least 1000 IU anti-D immunoglobulin given postpartum and anti-D offered after potentially sensitising events during pregnancy.
- Outcome: Sensitisation at 10 months postpartum

This protocol describes the intended population, intervention strategy, control strategy and outcome in the Trolle study, according to the published study report [15]. The actual study differed from the imagined idealised study in several respects, which will be discussed later in the paper.

To identify internal biases in the 10 studies of the effectiveness of RAADP, we compared each study against its idealised version. An internal bias checklist was completed for each study. This entailed answering a series of questions about potential sources of bias, and summarising relevant details extracted from the original papers. Following the methodology of Turner et al. [19], we considered the following five categories of internal bias: biases caused by differences between intervention and control groups at baseline (“selection bias”); biases related to lack of blinding of participants or caregivers (“performance bias”); biases caused by exclusions and drop-outs (“attrition bias”); biases related to measurement of the outcome (“outcome bias”) and additional biases (“other bias suspected”).

To identify external biases, we compared the idealised versions of the RAADP studies against the target setting. An external bias checklist was completed for each study. We considered the following four categories of external bias: biases caused by differences between the idealised study population and target population of non-sensitised pregnant Rhesus negative women in the UK (“population bias”); differences between the study and target interventions (“intervention bias”); differences between the study and target control strategies (“control bias”); and differences between the outcome measured in the study and the target outcome, sensitisation which would affect a subsequent pregnancy (“outcome bias”).

### Bias assessment

**Figure 1:**
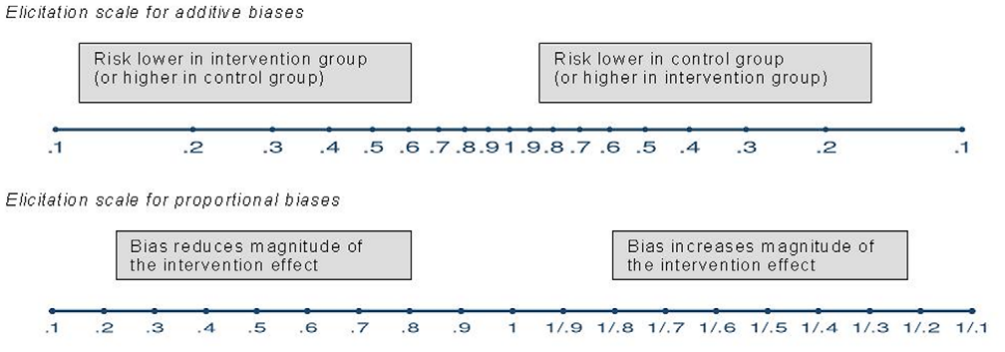
Elicitation scales. Elicitation scales for quantifying additive biases on a relative risk scale and for quantifying proportional biases as proportional change to (log) relative risk.

Internal biases were assessed by four assessors with quantitative expertise (three biostatisticians and one obstetrician), and external biases were assessed separately by four assessors selected for their knowledge of anti-D prophylaxis. At the elicitation meetings, the assessors discussed each study in turn and reviewed the bias checklist, while discussing any queries and resolving misunderstandings. For each bias, assessors agreed whether the bias would change only the magnitude of the intervention effect (a proportional bias) or whether it could change the direction of effect (an additive bias). After the group discussion, the assessors independently gave their opinions on the magnitude (and uncertainty) of each bias in each study, by marking ranges on bias elicitation scales (Figure 1). Opinions about the size and uncertainty in the biases were marked using 67% intervals, such that the assessor believed the true bias was twice as likely to lie inside rather than outside this range. For example, if the assessor believed that a study with historical controls was likely to be subject to a major additive internal selection bias favouring RAADP, then a 67% interval might be indicated as 0.4 to 0.8 on the left-hand side of the upper scale in Figure 1. If the assessor believed that a study of primigravidae was almost entirely relevant to all non-sensitised pregnant women, then an interval of 0.9 to 1/0.9 might be indicated for a proportional external population bias on the lower scale.

### Bias-adjusted meta-analysis

For each assessor, we calculated means and variances for total bias in each study, for additive and proportional biases separately. These were used to adjust the study effect estimates and standard errors for bias, while acknowledging the uncertainty about the extent of bias [19]. For each study, results were pooled across assessors by taking the medians of the assessors' adjusted estimates and standard errors, in order to obtain the opinion of a “typical” assessor [20]. Finally, a conventional random-effects meta-analysis was used to combine the bias-adjusted results across studies. Statistical heterogeneity was assessed using the *I^2^* statistic [21], which gives the percentage of variation between the study estimates attributable to true between-study heterogeneity rather than random variation; 0% indicates no heterogeneity.

### Analysis comparing different dose regimens

In a meta-regression analysis, we used the data available from the RAADP studies to inform us about the relative effectiveness of three anti-D dose regimens which were of particular interest in the second NICE appraisal. These are the three licensed anti-D treatments: 500 IU at 28 and 34 weeks; 1500 IU at 28–30 weeks; 1250 IU at 28 and 34 weeks. Four of the RAADP studies evaluated the effectiveness of the first of these [10], [12]–[14], but no studies have evaluated either of the latter two treatments exactly as specified. In principle, it would be possible to use the available study data to compare the effectiveness of giving 500 IU at 28 and 34 weeks with the effectiveness of giving 1500 IU at 28 weeks (evaluated in three studies [7], [8], [15]) or 1500 IU at 28 and 34 weeks (evaluated in one study [6]). However, the small number of studies means that differences between dose regimens would be very imprecisely estimated. Subgroup analyses comparing the effects of different doses cannot provide any conclusive findings in this data set.

We therefore elicited opinion on the relative effectiveness of all RAADP treatment regimens of relevance, comprising the five treatments evaluated in one or more studies and the two treatments which are licensed but as yet unevaluated. For each treatment regimen, four assessors with knowledge of anti-D prophylaxis were asked to provide numerical 67% ranges to describe their belief and uncertainty about the effectiveness of this treatment relative to an imagined optimally effective treatment (i.e. one which would prevent all sensitisations during pregnancy). Assessors were asked to take into account the half-life of prophylactic anti-D (approximately 3 weeks), the minimum circulating level required to provide protection against sensitisation, and anticipated compliance with each treatment regime. Pilgrim et al. [5] noted that compliance may be greater for a single-dose regimen for logistical reasons, but also that a two-dose regimen offers an opportunity to reduce the risk somewhat if the first appointment is missed.

Values on the elicitation scale for relative effectiveness ranged from 0 for a treatment no better than control to 1 for an optimally effective treatment. A meta-regression analysis [22] was performed to estimate the association between the measure of relative effectiveness (as a predictor measured with uncertainty) and the observed effectiveness of the five treatments on which data are available. The observed odds ratios were first adjusted for all internal and external biases except Intervention bias. The fitted model was then used to predict the underlying effectiveness odds ratio expected in a future study of any of the seven treatments, including the two on which no direct data are available.

## Results

### Study characteristics and extracted results

The designs of the ten RAADP studies included in our analyses are summarised in Table 1. The anti-D prophylaxis regimen administered to women in the intervention group varied across studies. Anti-D was given on either one or two occasions, and doses and planned timing of routine prophylaxis differed. There was also substantial variation in the time at which women were followed up for sensitisation to Rhesus positive antibodies. Sensitisation can be most accurately assessed in a subsequent pregnancy [5], as in the MacKenzie [12] and Mayne [13] studies, but some studies assessed sensitisation immediately after each woman's first delivery or at 6–12 months postpartum. The study populations varied somewhat across studies in terms of both their geographical locations, and the fact that some studies included non-sensitised multigravidae as well as primigravidae.

**Table 1:** Summary of characteristics of studies comparing RAADP to control, with reported odds ratios for sensitisation.

Study	Population	Intervention	Control	Outcome	OR (95% CI)
Bowman (1) [6]	Non-sensitised Rh− women delivered of Rh+ babies in a Canadian province	Two doses of 1500 IU anti-D Ig, given at 28 and 34 weeks' gestation, in addition to control care	1500 IU anti-D Ig given within 72 hours after delivery of a Rh+ baby or abortion	Sensitisation during pregnancy or within 3 days after delivery	0.02 (0.001, 0.33)
Bowman (2) [7]	Non-sensitised Rh− women delivered of Rh+ babies in a Canadian province	One dose of 1500 IU anti-D Ig, given at 28 weeks' gestation, in addition to control care	1500 IU anti-D Ig given within 72 hours after delivery of a Rh+ baby or abortion	Sensitisation at delivery	0.34 (0.18, 0.65)
Bowman (3) [8]	Non-sensitised Rh− women delivered of Rh+ babies in a Canadian province	One dose of 1500 IU anti-D Ig, given at 28 weeks' gestation, in addition to control care	1500 IU anti-D Ig given within 72 hours after delivery of a Rh+ baby or abortion	Sensitisation at delivery	0.18 (0.12, 0.28)
Hermann [9]	Non-sensitised Rh− women delivered of Rh+ babies at a Swedish hospital	One dose of 1250 IU anti-D Ig, given at 32–34 weeks' gestation, in addition to control care	1250 IU anti-D Ig given within 72 hours after delivery of a Rh+ baby	Sensitisation at 8 months postpartum	0.24 (0.05, 1.10)
Huchet [10]	Rh− primiparae delivered of Rh+ babies in 23 Parisian maternity units	Two doses of 500 IU anti-D Ig, given at 28 and 34 weeks' gestation, in addition to control care	500 IU anti-D Ig given after delivery of a Rh+ baby, repeated if necessary	Sensitisation at 2–12 months postpartum	0.14 (0.02, 1.14)
Lee [11]	Rh− primigravidae delivered of Rh+ babies in several UK obstetric units	Two doses of 250 IU anti-D Ig, given at 28 and 34 weeks' gestation, in addition to control care	Anti-D Ig given after delivery and after potentially sensitising events “in the usual way” (UK, 1992), doses not stated	Sensitisation at 6 months postpartum	0.56 (0.14, 2.24)
MacKenzie [12]	Non-sensitised Rh− primiparae delivered of Rh+ babies in two UK counties	Two doses of 500 IU anti-D Ig, given at 28 and 34 weeks' gestation, in addition to control care	“Standard” anti-D prophylaxis given after delivery and after potentially sensitising events (UK, 1990–1996), doses not stated	Sensitisation during second pregnancy	0.44 (0.22, 0.86)
Mayne [13]	Rh− primigravidae delivered of Rh+ babies in Derbyshire, UK	Two doses of 500 IU anti-D Ig, given at 28 and 34 weeks' gestation, in addition to control care	No information provided on control care; study setting was UK, 1988–1990	Sensitisation during second or subsequent pregnancy	0.25 (0.08, 0.74)
Tovey [14]	Rh− primigravidae delivered of Rh+ babies in Yorkshire, UK	Two doses of 500 IU anti-D Ig, given at 28 and 34 weeks' gestation, in addition to control care	500 IU anti-D Ig given after delivery of a Rh+ baby, or a higher dose if the Kleihauer count was abnormal	Sensitisation in a subsequent pregnancy	0.16 (0.04, 0.67)
Trolle [15]	Non-sensitised Rh− women delivered of Rh+ babies at a Danish hospital	One dose of 1500 IU anti-D Ig, given at 28 weeks' gestation, in addition to control care	1000 IU anti-D Ig given after delivery of a Rh+ baby	Sensitisation at 10 months postpartum	0.08 (0.005, 1.49)

Table 1 presents the odds ratios extracted from each study, comparing RAADP to control. Six of the studies found evidence of a lower sensitisation rate in women who received RAADP. Odds ratio estimates in the other four studies were also well below 1, but the associated confidence intervals were wide as a consequence of small sample sizes. Substantial differences in design mean that a simple meta-analysis would be inappropriate, and the many methodological limitations in the studies would make interpretation problematic. We addressed these issues by performing a bias-adjusted meta-analysis.

### Biases identified

The potential internal biases affecting each of the 10 studies, as identified through completion of an internal bias checklist, are summarised in Table 2. Selection bias was a major concern in seven studies, since women receiving RAADP were compared to historical controls who were very likely to have differed from the intervention women in many respects, and the statistical analyses did not adjust for confounding. The lack of blinding (which would be difficult in this setting for ethical reasons) of both subjects and caregivers means that the chance of receiving directed anti-D after a potentially sensitising event possibly differed between intervention and control groups in all studies. In addition, there were losses to follow-up, although not all studies provided information on this, and there were problems with assessing sensitisation accurately in a few of the earliest studies.

**Table 2:** Potential internal biases identified in the studies.

Study	Selection bias	Performance bias	Attrition bias	Outcome bias
Bowman (1) [6]	Historical controls used, and location of women recruited also differed to some extent between groups. Confounding not addressed.	No blinding, so the likelihood of receiving directed anti-D after potentially sensitising events may differ between groups.	Unclear – losses to follow-up not reported.	Suggestion that outcome assessors couldn't distinguish between immune and passive antibodies.
Bowman (2) [7]	Historical controls used in comparison between groups. Confounding not addressed.	No blinding, so the likelihood of receiving directed anti-D after potentially sensitising events may differ between groups.	Unclear – losses to follow-up not reported.	Suggestion that outcome assessors couldn't distinguish between immune and passive antibodies in control group.
Bowman (3) [8]	Historical controls used in comparison between groups. Confounding not addressed.	No blinding, so the likelihood of receiving directed anti-D after potentially sensitising events may differ between groups.	Unclear – losses to follow-up not reported.	Suggestion that outcome assessors couldn't distinguish between immune and passive antibodies in control group.
Hermann [9]	Historical controls used, and very little information on inclusion/exclusion criteria. Confounding not addressed.	No blinding, so the likelihood of receiving directed anti-D after potentially sensitising events may differ between groups.	Some posthoc exclusions in intervention group. No information on losses to follow-up or exclusions in control group.	Suggestion that outcome assessors couldn't distinguish between immune and passive antibodies.
Huchet [10]	Contemporary controls used, but allocation to groups was non-random. Confounding not addressed.	No blinding, so the likelihood of receiving directed anti-D after potentially sensitising events may differ between groups.	4% of women lost to follow-up after recruitment. No outcome available for 21% of remaining treated women and 21% of controls.	
Lee [11]		No blinding, so the likelihood of receiving directed anti-D after potentially sensitising events may differ between groups.	21% of treated women and 16% controls lost to follow-up after recruitment. No outcome available for 32% of remaining treated women and 38% of controls.	
MacKenzie [12]	Contemporary controls used, but location of women recruited differed between groups. Confounding not addressed.	No blinding (as above). No individual follow-up, so women migrating into intervention group area may not have received routine anti-D in first pregnancy.	Unclear – insufficient details on how sensitisation rates in the two groups were determined.	
Mayne [13]	Historical controls used. Confounding not addressed.	No blinding (as above). No individual follow-up, so women migrating into intervention group area may not have received routine anti-D in first pregnancy.	Unclear – insufficient details on how sensitisation rates in the two groups were determined.	
Tovey [14]	Historical controls used. Confounding not addressed.	No blinding (as above).	Unclear – losses to follow-up not reported.	
Trolle[15]	Historical controls used. Treated group excludes sensitised women at 28 weeks, but control group does not. Confounding not addressed.	No blinding (as above). In addition, some controls may not have received anti-D after delivery, as control period predates routine use of anti-D in Denmark.	16% of treated women and 9% controls lost to follow-up.	Possibility that outcome assessors couldn't distinguish between immune and passive antibodies.

External biases arose from differences between the available RAADP studies and the pre-defined target setting with respect to population, intervention, control strategy and the outcome measure (Table 1).

### Bias-adjusted results for one study

**Figure 2:**
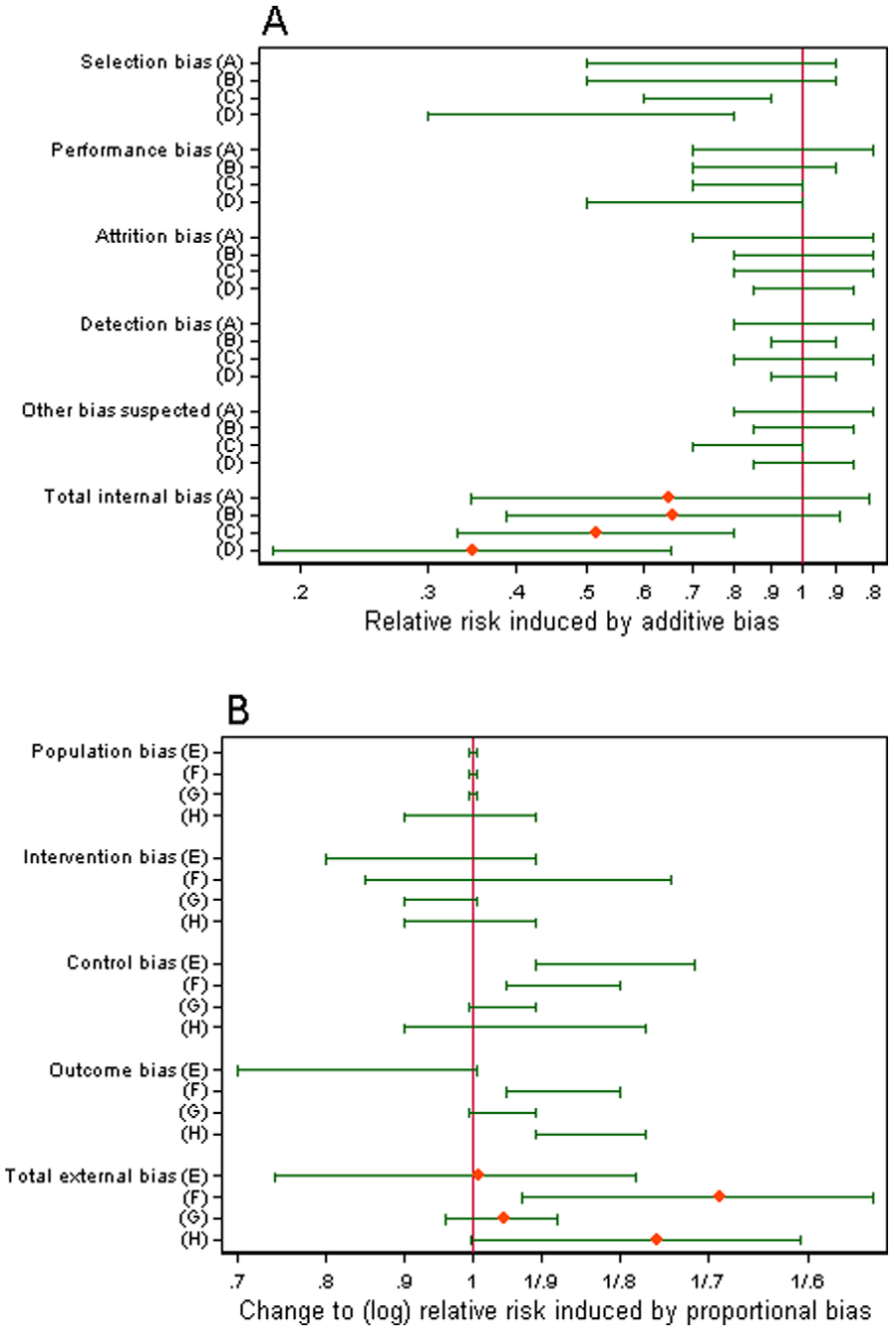
Biases in the Trolle study. (a) 67% ranges elicited from assessors A–D for additive internal biases, with means and 67% ranges for total internal bias; (b) 67% ranges elicited from assessors E–H for proportional external biases, with means and 67% ranges for total external bias.

To exemplify the methods, we first discuss bias adjustment in the Trolle study [15]. The bias assessments provided by individual bias assessors for internal and external biases in this study are presented in Figure 2. In this study, all internal biases were regarded as additive and all external biases were regarded as proportional. Two of the biases (selection bias and performance bias) were expected by all assessors to make the intervention appear more effective. Selection bias was caused by use of historical controls and inclusion policies which differed between the intervention and control groups (Table 2). Performance bias was caused by lack of blinding and the possibility that some women in the control group did not receive anti-D postpartum. There was a general degree of consistency among the four assessors' opinions on the internal biases.

There was more variation in assessors' opinions on the external biases. Most assessors were uncertain whether the difference between the intervention evaluated by Trolle and the target intervention would cause the intervention effect to be exaggerated or reduced. All assessors believed that the difference between antenatal care received by the control group in the Trolle study and antenatal care in the target control setting would lead to an exaggerated effect. By measuring the outcome at 10 months postpartum, the Trolle study may have failed to detect some sensitisations which would only become obvious in a subsequent pregnancy. Three assessors expected that this was likely to cause an exaggerated effect in the Trolle study relative to a study measuring the target outcome of sensitisation in a subsequent pregnancy, while one assessor expected a reduced effect.

**Figure 3:**
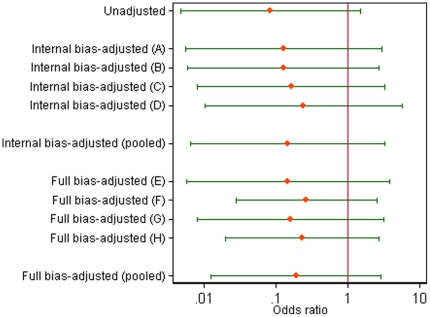
Impact of adjusting for internal and external bias in the Trolle study. Impact of adjusting initially for internal bias and subsequently for both internal and external bias (using pooled internal bias-adjusted results) on the odds ratio (and 95% interval), for each assessor separately and combined using median pooling.

The impact of adjusting the Trolle results for (a) internal biases and (b) external biases is shown in Figure 3. Since internal biases were believed to have favoured the intervention group, the bias-adjusted odds ratio has shifted towards the null value 1. External biases were generally expected to have caused an exaggerated effect in the Trolle study compared to the target setting, so adjustment for these also caused a shift towards 1. The width of the 95% confidence interval for the odds ratio was not much affected by the adjustment for bias and its uncertainty, because the unadjusted estimate was already imprecise in this study.

### Bias-adjusted meta-analysis results

In a bias-adjusted meta-analysis of the ten RAADP studies, the pooled odds ratio for sensitisation was estimated as 0.31 (95% CI 0.17, 0.56), with no evidence of heterogeneity (*I* ^2^ = 0%). This result provides strong evidence for the effectiveness of RAADP in preventing sensitisation of pregnant Rhesus negative women. We compare this result with the clinical effectiveness findings in the two NICE appraisals [4], [5], which did not include a quantitative synthesis of all evidence. In both NICE appraisals, the principal clinical findings were based on a fixed effect meta-analysis of two studies, MacKenzie [12] and Mayne [13], which were considered to be most relevant to the UK setting and of higher quality. Pooling the results of these two studies (with no adjustment for bias) gave an odds ratio for sensitisation of 0.37 (95% CI 0.21, 0.65). This is similar to the result from the bias-adjusted meta-analysis including all 10 studies, and we note that the MacKenzie and Mayne studies received highest weight in our analysis, 31% and 14% respectively.

**Figure 4:**
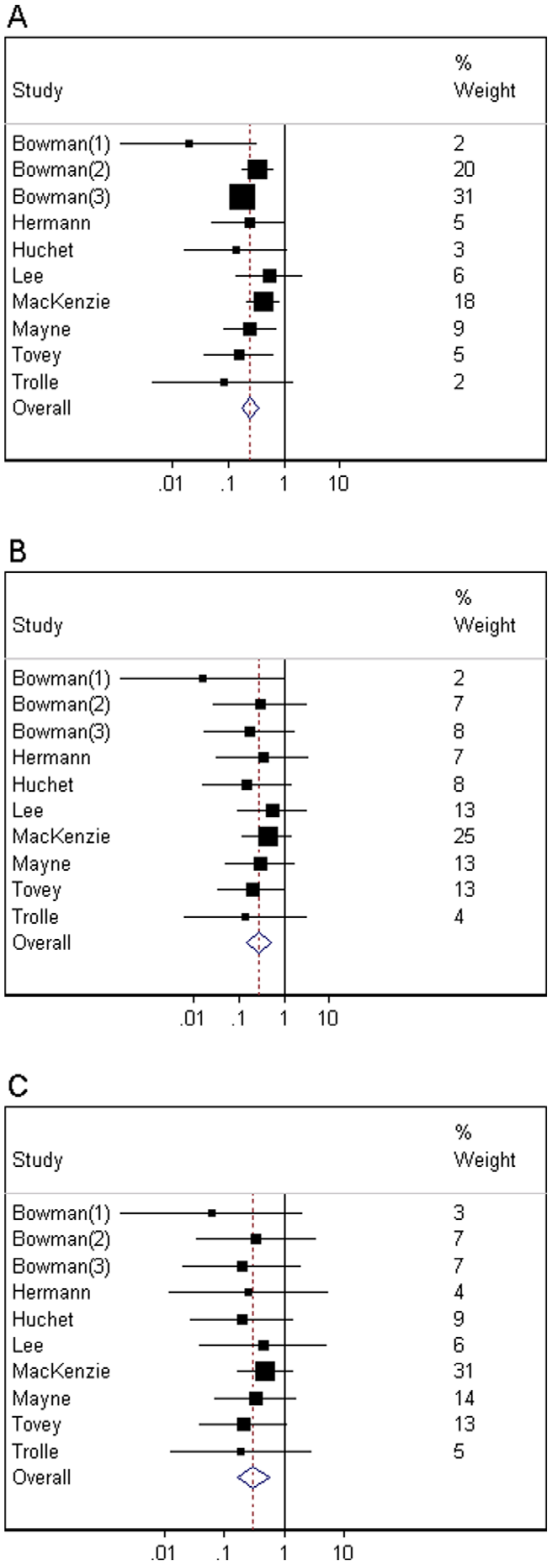
Impact of adjusting for bias in the meta-analysis of 10 studies comparing RAADP to control. (a) unadjusted odds ratios (with 95% CIs); (b) odds ratios adjusted for internal biases (with 95% CIs); (c) odds ratios adjusted for all biases (with 95% CIs).

To illustrate the impact of our bias adjustments on the results of the 10 studies, Figure 4 presents three versions of the meta-analysis: first unadjusted, secondly adjusted for internal biases and thirdly adjusted additionally for external biases. After adjusting for internal and external biases, all but one of the study estimates shifted towards the null value 1, and most confidence intervals widened to reflect increased uncertainty about results affected by biases. A naïve conventional random-effects meta-analysis of all 10 studies produced a pooled odds ratio of 0.25 (95% CI 0.18, 0.36), comparing RAADP to control, with some heterogeneity (*I* ^2^ = 19%). This result does not acknowledge the uncertainty caused by biases, so the confidence interval is inappropriately narrow. After adjusting for internal biases, the pooled odds ratio for sensitisation was estimated as 0.28 (95% CI 0.15, 0.53), comparing RAADP to control, and there was no remaining evidence of between-study heterogeneity (*I* ^2^ = 0%). This result allows for the methodological limitations in the RAADP studies, but does not acknowledge their varying relevance to the target setting of interest to NICE.

As a sensitivity analysis, we also present results from a meta-analysis excluding the Bowman (2) and Bowman (3) studies [7], [8], which share a control group with Bowman (1) [6]. In a bias-adjusted meta-analysis of these 8 studies, the pooled odds ratio for sensitisation was estimated as 0.31 (95% CI 0.16, 0.61), with no evidence of heterogeneity (*I* ^2^ = 0%). The numerical result is similar to that based on all 10 studies and the conclusions from the analysis are unchanged. To check the robustness of our findings, we also performed separate meta-analyses using the opinions of each assessor in turn. The results obtained from adjusting for the opinions of each internal bias assessor were similar to each other and close to results adjusted for pooled opinion on internal bias. Meta-analyses adjusted for each external bias assessor in turn (following adjustment for pooled opinion on internal bias) were also similar to each other and close to the pooled bias-adjusted meta-analysis.

### Analysis comparing different dose regimens

**Figure 5:**
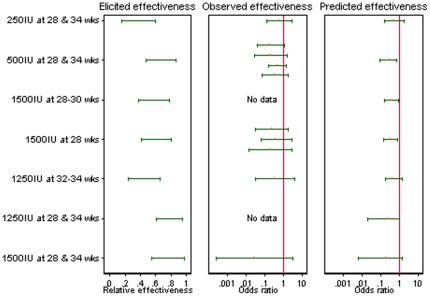
Exploratory analysis comparing different dose regimens. For each of seven RAADP treatment regimens: elicited relative effectiveness compared to an optimally effective treatment (67% intervals pooled across assessors); observed odds ratios comparing RAADP to control (with 95% intervals), where available; and odds ratios expected in a future study comparing RAADP to control (with 95% intervals), obtained from a fitted meta-regression model. (Higher values for relative effectiveness compared to an optimal treatment correspond to lower odds ratios compared to control.).

In a meta-regression analysis, we estimated the association between the observed results for each anti-D dose in the studies available and the pooled opinion on a measure of relative effectiveness of each dose (Figure 5). Within this analysis, we predicted the underlying odds ratios we would expect for each of the three licensed anti-D treatments, in a new study evaluating their effectiveness compared to control (without allowance for sampling variation). The estimated odds ratios for sensitisation are 0.31 (95% CI 0.09, 0.65) for a dose of 500 IU at 28 and 34 weeks, 0.42 (95% CI 0.17, 0.73) for a dose of 1500 IU at 28–30 weeks, and 0.18 (95% CI 0.03, 0.53) for a dose of 1250 IU at 28 and 34 weeks. Using the data available for other doses of anti-D and the beliefs on relative effectiveness elicited from four assessors, we have estimated odds ratios for the effectiveness of two licensed treatments which have not yet been evaluated. Each of the two unevaluated treatments is expected to be effective for prevention of sensitisation. Among the three licensed treatments, the estimated probability that a dose of 1250 IU at 28 and 34 weeks is most effective is 83%, while the probability that a dose of 500 IU at 28 and 34 weeks is most effective is 15%. The probability that a single dose of 1500 IU at 28–30 weeks is least effective amongst these three regimens is estimated as 76%.

## Discussion

A bias-adjusted meta-analysis allowed us to synthesise all available evidence on the effectiveness of RAADP, while adjusting for differences in study quality and design. The pooled bias-adjusted results presented here provide strong evidence that RAADP prevents sensitisation in pregnant Rhesus negative women. These findings confirm the conclusions of the two NICE appraisals, which did not include a quantitative synthesis of all evidence. It is reassuring to find that, when all evidence on clinical effectiveness is taken into account, the widespread policy of offering RAADP to all non-sensitised pregnant Rhesus negative women remains strongly supported. However, the cost-effectiveness of this policy should also be reconsidered. Since the bias-adjusted meta-analysis of 10 studies produces a larger treatment effect estimate (and 95% interval further from the null value) than the meta-analysis of two studies used in cost-effectiveness modelling in the NICE appraisals, it is very likely that RAADP would again be judged cost-effective on the basis of the new results.

Two of the licensed anti-D treatments have not yet been evaluated. In a meta-regression analysis, we predicted the underlying effectiveness odds ratios that we would expect for these treatments if they were compared against control antenatal care in a future trial. In the absence of a head-to-head trial comparing the three licensed treatments directly, this provides useful information on their expected relative effectiveness. Our model gave a high probability that a dose of 1250 IU at 28 and 34 weeks would be the most effective amongst the three licensed treatments, and a high probability that a single dose of 1500 IU at 28–30 weeks would be the least effective. These results could inform an economic model to compare the expected cost-effectiveness of the three licensed treatments and determine which is the optimum dose regimen of RAADP. However, a large randomised trial comparing the three licensed doses would provide much firmer evidence on their relative clinical effectiveness and cost-effectiveness.

When comparing the licensed regimens, we have not taken into account ethical considerations. Some women are reluctant to receive blood products and their reluctance may be greater for a two-dose regimen using larger doses of immunoglobulin. If problems with availability of anti-D immunoglobulin are encountered in the future, a regimen which minimises the total volume of plasma administered would be preferred. New technologies currently under development could potentially lead to considerable changes in the antenatal care of Rhesus negative women. Recombinant anti -D immunoglobulin is expected to become available and would provide a safer, more acceptable alternative to human plasma immunoglobulin. In addition, if antenatal fetal genotyping becomes widespread and cost-effective, this would allow targeted antenatal anti-D prophylaxis in pregnancies where the fetus is found to be Rhesus positive [23].

Without adjustment for variations in study design and quality, it would be inappropriate to combine evidence from all available RAADP studies. In comparison with the unadjusted meta-analysis, the weights given to the studies in calculation of the pooled result changed considerably after adjustment for biases. In particular, the weights given to the higher quality studies (MacKenzie [12], Mayne [13] and Huchet [10]) were increased, while the influence of lower quality studies was reduced. By adjusting for bias and acknowledging uncertainty over its impact, we aimed to remove unexplained between-study heterogeneity from the meta-analysis [19], and this was achieved in the analysis of the RAADP studies.

The bias-adjustment process relies on incorporating expert opinion. Subjective judgement is routinely used in meta-analysis. The standard approach to handling studies of diverse quality and design is to choose a minimum threshold for inclusion and regard those studies included as unbiased. By presenting results from both unadjusted and bias-adjusted meta-analyses, as in this paper, subjective opinion is made transparent and accountable. The experts who quantified the biases were carefully chosen for their knowledge of anti-D prophylaxis (or their quantitative expertise, for internal biases), and their opinions were combined in such a way that no individual view could overly influence the final results of the meta-analysis.

### Conclusion

A bias-adjusted synthesis of all available evidence provides strong evidence for the effectiveness of RAADP in preventing sensitisation, in support of the policy of offering RAADP to all non-sensitised pregnant Rhesus negative women. All three licensed RAADP treatments are expected to be effective in preventing sensitisation. A dose of 1250 IU at 28 and 34 weeks is expected to be most effective and a single dose of 1500 IU at 28–30 weeks is expected to be least effective.
